# A revision of *Artemia* biodiversity in Macaronesia

**DOI:** 10.1186/2046-9063-8-25

**Published:** 2012-10-18

**Authors:** Francisco Hontoria, Stela Redón, Marta Maccari, Inmaculada Varó, Juan Carlos Navarro, Lluis Ballell, Francisco Amat

**Affiliations:** 1 Instituto de Acuicultura de Torre de la Sal (CSIC), 12595 Ribera de Cabanes, Castellón, Spain

**Keywords:** *Artemia*, Biodiversity, Macaronesia, Saltworks, Loss of habitats, Invasion

## Abstract

In a biogeographical context, the term Macaronesia broadly embraces the North Atlantic archipelagos of the Azores, Madeira, Selvagens, the Canary Islands, and Cape Verde. The peculiar arid climatic conditions in some of these places have led to the development of marine salt exploitations, which can be counted among the hypersaline habitats of the brine shrimp *Artemia* (Branchiopoda, Anostraca). Parthenogenetic populations of this anostracan were described in the Canary Islands during the last decades of the 20th century, while the American *Artemia franciscana* species was recently found in the Cape Verde archipelago. Following an invasive pattern, this exotic species has recently reached the Canary Islands, too. This paper reports information dealing with biotope loss (solar saltworks) in this biogeographical region, together with possible consequences concerning the arrival of invasive species, two factors that frequently promote dramatic biodiversity losses. The discussion of this threat focuses mainly on the Canary Islands archipelago where native species of *Artemia* still exist.

## Macaronesian archipelagos

The Macaronesian biogeographic region mainly comprises an array of volcanic islands in the Atlantic Ocean. The term Macaronesia was coined by Engler [1] who recognized the unit as comprising only the Azores, Madeira, Selvagens and Canary Archipelagos. Later authors included the Cape Verde archipelago in the Macaronesian circumscription [2], and the unit was biogeographically enlarged with the inclusion of continental areas of North Africa and the Iberian Peninsula.

The Macaronesian islands are characterized by very steep landscapes, with the Teide volcano in the island of Tenerife in the Canaries reaching an altitude of 3,718 m. Because of their volcanic origin, it used to be said that these archipelagos were never attached to the African continent. However, it has recently been shown [3] that several volcanic sea mountains located between the African continent, the Canaries Archipelago, the Selvagens Islands and Madeira, and today less than 100 m below sea level, are at least 68 MY old, so they may once have formed a chain of islands facilitating the migration of species between the archipelagos and Africa.

The four archipelagos differ substantially in terms of topography, geomorphology and climate. The Azores and Madeira, together with the western Canary Islands (La Palma, El Hierro, Gomera) show oceanic island landscapes, while the eastern Canary Islands, together with the Cape Verde Archipelago are dominated by desert landscapes very similar to those found in the neighbouring African continent.

The Azores Archipelago consists of nine clearly oceanic islands. They are the youngest part of Macaronesia, with a landscape formed by river valleys in eroded volcanic rocks. The predominant winds from the north-west create a temperate humid climate (14–22°C) with high rainfall.

Madeira consists of three oceanic islands, approximately 700 km from Africa and 950 km from Europe. Its eastern part is very steep, while the western part is lower and hosts a high plateau. It is also rainy with a warm-temperate climate similar to that of the Canary Islands.

The Canaries Archipelago consists of seven main islands: La Palma, El Hierro, Gomera, Tenerife, Gran Canaria, Lanzarote and Fuerteventura, this last being 96 km from Cape Jubi in southern Morocco. All the islands show very rugged landscapes as a result of recent volcanic activities. The climate is warm-temperate, the mean temperature ranging between 20 and 22°C all the year round. Rainfall is low, especially in the dry and lower eastern islands of Lanzarote and Fuerteventura, with a mean of 100–150 mm per year.

The Cape Verde is the most arid archipelago in African Macaronesia, being 500 km from the coasts of Senegal. The archipelago consists of ten islands divided into three groups: the north western islands of Santo Antão, San Vicente, Santa Luzia and San Nicolau; the eastern islands of Sal, Boa Vista and Maio; and the southern islands of Santiago, Fogo and Brava. Among them, those forming the eastern group, together with Santa Luzia, are the older islands, and have been subjected to erosional agents for longer periods, showing a low relief with scattered peaks [4] and extensive coastal flat lands. The climate is hot and desert-like, with temperatures reaching 30°C. Rainfall is scarce and irregular.

## Hypersaline biotopes in Macaronesia

The brine shrimp *Artemia* (Crustacea, Branchiopoda, Anostraca) is a cosmopolitan organism inhabiting hypersaline natural ecosystems (salt lakes and lagoons), as well as man made solar salterns built for commercial salt exploitation. These ecosystems usually show low biodiversity values because the conditions are too harsh for the development of living organisms.

Human occupation of the Macaronesian archipelagos, their topography and climate led to the installation of marine solar saltworks in the Canary Islands and Cape Verde, but not in Madeira or the Azores. It is reported that the Romans reached Lanzarote and La Graciosa islands in search of salt extracted from sea shore rocky pools and from the briny lagoon present in El Rio (29º13’86”N – 13º29’24”W), which was converted into a solar saltern in the 15th century.

In the Canary Islands, saltworks have been recorded on all islands with the exception of La Gomera [5]. A summary of their number, surface area and age is presented in Table 1. Only two saltworks were recorded from the western oceanic islands of La Palma and El Hierro, one in each. These were small, only a few hundred m^2^ in El Hierro and scarcely 2 ha in La Palma. Surprisingly, the Fuencaliente saltworks (28º27’15”N–17º50’28”W) in La Palma are the westernmost and also the newest in the archipelago. Extraction began in 1971 but had to be suspended in 1972 because of the Teneguia volcano eruption; however, they have been in operation since then.

**Table 1:** Inventory of old saltworks reported in Canary Islands and Cape Verde archipelagos, and those still working today

**Archipielago**	**Island**	Saltworks reported (*)	**Mean area (ha)**	**Saltworks still working**	**Name**	**Year starting activity**	**Annual salt production (t)**
Canary Islands	La Palma	2	1.25	1	*Fuencaliente*	1967	420
	El Hierro	2	0.04	0			
	Tenerife	5	1.80	0			
	Gran Canaria	12	1.50	3	*Tenefé*	1800	200
					*Boca Cangrejo*	1800	110
					*Puerto Arinaga*	1790	400
	Fuerteventura	4	3.20	1	*El Carmen*	1910	400
	Lanzarote	27	7.00	2	*Janubio*	1915	13,000
					*Guatiza*	1940	500
	**Total**	**52**		**7**			
Cape Verde	Sal	3	31.00	1	*Pedra Lume*	?	?
	Boa Vista	3		0			
	Maio	2		0			
	**Total**	**8**		**1**			

(*) Modified and summarized from Marin and Luengo [5].

Saltworks in the other Canary Islands are more numerous and extensive, with a mean area of between one and three ha in Tenerife, Gran Canaria and Fuerteventura. In Lanzarote 27 saltworks have been recorded, with a mean surface area of 7 ha each, El Rio being the oldest and Janubio (28º56’24”N – 13º49’18”W) the biggest with more than 43 ha in continuous operation from 1915.

Most of the biggest and more productive saltworks are associated with the existence of natural coastal lagoons. They are filled with sea water from high tides or using pumping devices (wind mills or modern pumps), and act as a sea water reservoir or brine evaporator. This is the case, for example, with El Rio and Janubio saltworks in Lanzarote.

Solar saltworks in Cape Verde are most commonly found in Sal, Boa Vista and Maio islands. Old salterns in Boa Vista and Maio are also subsidiary to natural sea shore lagoons. The most conspicuous saltworks are located in Pedra Lume (Sal Island) (16º46’07”N – 22º53’49”W) where the endorrheic sediments accumulated at the bottom of a volcanic crater provide the waterproof bed for the marine salt exploitation.

## The presence of *Artemia* populations in Macaronesia

The cosmopolitan genus *Artemia* currently comprises seven bisexual species, and a variety of parthenogenetic (clonal) lineages of different ploidy [6]. Two of these bisexual species are native from America: *A. franciscana* (Kellogg 1906), which has spread all around the American continent, and in South America, and *A. persimilis* (Piccinelli & Prosdocimi 1968), which is nearly exclusive in the American South Cone [7]. In the Old World, biogeography points to the dominance of bisexual species.

*A. salina* (Linnaeus 1758) inhabits the Mediterranean Basin, while *A. urmiana* (Gunther, 1900) is present in Iran and the Crimea [8,9], *A. sinica* (Cai 1989) is found in China and neighbouring provinces, *A. tibetiana* (Abatzopoulos, Zhang and Sorgeloos 1998) is present in salt lakes of the Tibetan plateau, and an undescribed population of *Artemia* sp. has been reported in Kazakhstan [10]. Several clonal lineages described with the binomen *Artemia parthenogenetica* (Bowen and Sterling 1978) are found in the whole Old World and Australia. While the bisexual species in the Old World remain more or less restricted in their distribution to the areas referred to above, the parthenogenetic lineages dispersed through the Eurasian and African continents, and have been recorded from Japan [8] to the Canary Islands [11] and South Africa [12].

**Figure 1:**
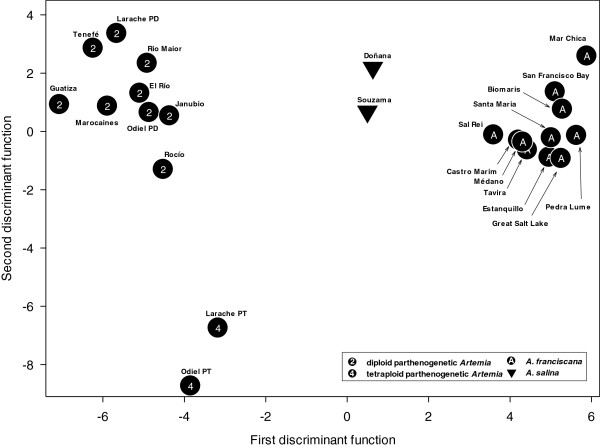
**Group centroids of the populations studied for the first two discriminant functions resulting from the discriminant analysis on female morphometric variables.** Macaronesian *Artemia* populations: Guatiza, El Río, Tenefé, Janubio (*A. parthenogenetica*) and Santa María, Pedra Lume, Sal Rei, Médano (*A. franciscana*) were compared with other *Artemia* populations (5 diploid *A. parthenogenetica*, 2 tetraploid *A. parthenogenetica*, 2 bisexual *A. salina* and 5 bisexual *A. franciscana*) chosen for every native species from locations neighbouring the Macaronesian Islands in Spain, Portugal and Morocco [20,35]. American populations from San Francisco Bay (California, USA) and Great Salt Lake (Utah, USA) are used as reference populations for the invasive species *A. franciscana.*

The first report on *Artemia* in Macaronesia pointed to the presence of a diploid parthenogenetic population [11,13] in the Janubio saltworks, in Lanzarote (Canary Islands). This finding was subsequently supported [14] with the description of another diploid parthenogenetic population in Salinas del Castillo (also referred to as Salinas del Carmen) saltworks (28º22’16”N-13º52’13”W) in Fuerteventura (Canary Islands). *Artemia* was not prospected again in the Canary Islands until the last decade (2006–2010). *Artemia* cysts were sampled from Tenefé saltworks (27º48’39”N-15º25’22”W) in Gran Canaria in 2006, as well as from Janubio, Guatiza (29º03’40”N-13º27’41”W) and El Rio saltworks in Lanzarote in 2010. When the saltworks located in Fuencaliente (La Palma Island) were visited in 2009, neither live *Artemia* populations nor cysts were found there. However, *Artemia* cysts were obtained in 2009 from a temporary sea shore pool which becomes briny and dry in summer, located in El Médano area (28º02’08”N-16º32’37”W), in the southeast of the island of Tenerife. All these cyst samples were hatched in the laboratory, the nauplii obtained were grown to reach adulthood in standard culture conditions, and adult specimens were studied morphometrically for systematic classification according to the multivariate discriminant analysis method previously described [15]. The all-female *Artemia* populations from Tenefé, Guatiza and El Rio saltworks could clearly be adscribed to diploid parthenogenetic lineages, while a sexual *A. franciscana* population was obtained from the cysts collected in El Médano pool (Figure 1).

Information on the presence of *Artemia* populations in the hypersaline ecosystems from Cape Verde was not available until very recently. *Artemia* cysts were sampled [16] in 2005 from the Pedra Lume (16º46’07”N-22º52’50”W) and Santa Maria (16º33’23”N-22º53’59”W) saltworks in the Sal Island, and from Sal Rei (16º08’38”N-22º52’24”W) saltworks in Boa Vista Island. Morphometrical methods [15] allowed these populations to be classified as *A. franciscana*.

## The threat to *Artemia* biodiversity in the Canary Islands

The loss of environments suitable for the development of autochthonous species and invasion by exotic species are the basis of global biodiversity damage [17,18].

### Biotope loss

Hypersaline aquatic ecosystems, like coastal and epicontinental lagoons, salt lakes, even exploited solar saltworks are dramatically suffering from these impacts [19-21]. Many human activities threaten or have already damaged salt marshes and hypersaline ecosystems through inflow diversions, pollution, biological disturbances, and diverse anthropogenically-induced damage like abandonment in favour of other economical activities. Small solar saltworks managed according to old artisanal techniques are amongst the most threatened of these ecosystems. The data shown in Table 1 confirm that of about 60 saltworks reported from both archipelagos (52 in Canary Islands and 8 in Cape Verde), only 8 of these exploitations can be considered to be still working. Most are small, were established during the 18th and 20th centuries, occupy areas ranging between 1 and 3 ha, and are still managed in an artisanal way, producing between 110 and 500 metric tonnes of sea salt per year. The only exceptions are the new Fuencaliente saltworks in La Palma, and Janubio saltworks in Lanzarote. Janubio covers an area of about 44 ha and produces about 13,000 tonnes per year [5]. Most of these saltworks still persist because they lie in natural areas protected by government environmental agencies. This is the case of Fuencaliente and Janubio in the Canary Islands and Pedra Lume in Cape Verde, which still exist because of their natural landscape, biodiversity protection and tourist interest. Briefly, it can be asserted that biotope loss in terms of abandoned saltworks is about 85% in both archipelagos.

### Invasive species

It is not possible to assert the origin of the *A. franciscana* population found in El Médano sea shore pool (Tenerife, Canary Islands) and in the Pedra Lume, Santa Maria and Sal Rei salterns (Cape Verde), all far from the native distribution range of this American species. Most probably *A. franciscana* cysts reached these biotopes for deliberate commercial reasons (salt production) or through erroneous or inadvertent intrusive inoculations (pet trade). But, beyond question, these inoculations occurred at different moments in the past. As stated above, the presence of native diploid parthenogenetic *Artemia* populations in the Canary Islands has been known since the 1980’s, but no *Artemia* populations have been described in the western Canary Islands, i.e. in Tenerife, since then.

The presence of native *Artemia* in the Canary Islands can be closely related to the natural dispersion of *Artemia* cysts from Europe (Spain, Portugal) [11,13,14,22] or from the African continent, especially Morocco, where this strain was found in Salines Marocaines (32º53’50”N-9º50’20”W) and in the saltworks at Larache and Asilah (35º11’50”N-6º07’08”W) [20]. In the western Sahara, not more than 100 km away from Lanzarote and Fuerteventura islands, there are several hypersaline biotopes and saltworks, e.g., Dait Um Saad El Aaiun (27º09’52”N-13º11’44”W) and Sebhet Taazgha (27º32’59”N-13º00’31”W), which could be considered a hypothetical source of dispersion towards the Canary Islands, although there is no information about the presence or biodiversity of *Artemia* populations there.

The Cape Verde *A. franciscana* populations could have reached this archipelago earlier because they show an important level of genetic divergence compared with other American populations that invaded the western Mediterranean biotopes in Spain, Portugal, France, Morocco and Italy [23]. There is no information about the possible existence of native Old World *Artemia* populations in this archipelago before the arrival of *A. franciscana* as an exotic species.

Even considering the previous presence of native species as a result of a natural dispersion from the African continent, the nearest hypersaline biotope that could be the source of this dispersion is located in Senegal, in the Kaolack saltworks (14º06’54”N-16º05’37”W) about 750 km away from the easternmost Cape Verde saltworks (15º59’29”N-22º47’10”W) in Curral Velho (Boa Vista island). Nevertheless, there is no information available on the biodiversity of *Artemia* in Senegal, although some publications on the global geographical distribution of *Artemia* species mention the presence of at least three localities in this country where *Artemia* could be present: Dakar, Lake Kayar and Lake Retba. In these publications Kaolack saltworks are not cited. No additional information is available on the taxonomy of the species present in these locations or on their way of reproduction [12,23,24].

The presence of *A. franciscana* in El Médano could be the origin of the dispersion of this exotic species towards other hypersaline biotopes existing in the eastern Gran Canaria, Lanzarote and Fuerteventura Islands, as well as towards the west (La Palma). In addition to possible anthropic intrusive inoculation, the role of migratory and resident aquatic birds in spreading brine shrimp cysts, attached or stuck to feathers and feet, has been demonstrated [25]. They can also survive passage through the gut after capture as food by birds, and cysts may be excreted during displacements in search of food or during seasonal migrations [26]. This possibility has been broadly tested after verifying the presence of large numbers of viable cysts of invasive *A. franciscana* and native *A. parthenogenetica* in the faeces and pellets of common redshank (*Tringa totanus*)*,* blacktailed godwit (*Limosa limosa*) and dunlin (*Calidris alpina)* collected in saltworks in the south-west of the Iberian Peninsula, at Castro Marim (Portugal) and Cadiz Bay (Spain) [25,26]. Several species of these shore bird groups have been reported as regular winter visitors in the south of Tenerife (El Médano) and in other saltmarshes and saltworks in the eastern Canary Islands [27,28]. These sites probably play a role as stopover and feeding places for these birds as they migrate from the European continent to African stating areas along the East Atlantic flyway [25,26]. If this is so, the dispersion of brine shrimp cysts and their inoculation in new biotopes is almost guaranteed.

The probable arrival of *A. franciscana* cysts from the El Médano population to the other islands housing active saltworks and native diploid parthenogenetic *Artemia* populations would have triggered a competitive coexistence between both forms and, as is well known, parthenogenetic strain usually comes out as loser [20].

Knowledge of the mechanisms leading to the success of invasive species relies on our understanding of any interactions triggered between the exotic and the native species, particularly as regards the attributes of biological fitness characterizing both invaded and invasive species. Assessment of this biological fitness led to the study of life table parameters for species under laboratory experimental conditions, which provided the knowledge necessary to explain the results of competition between native species and congeneric invasive exotic species [29-31].

In the case of *Artemia,* much information is available on competitive interactions between different species and populations, and between sexual species and parthenogenetic strains [20,32-35]. Competitive superiority begs the classical questions referring to invasive species: (i) why do invasive exotic species colonize and displace native species that should be better adapted to local environments [36,37] and (ii) why do invasive species flourish despite reduced genetic diversity in the recipient region [37]?

*A. franciscana* in the Old World should display less genetic diversity than in its American native range. Founder events and population bottlenecks in the early stages of introductions are considered responsible for the loss of biodiversity of many invasive species. Genetic diversity for nuclear markers must be lower as a consequence of the founder effect during introduction. However, in the case of *A. franciscana*, and as regards its introduction into the Macaronesian islands (Tenerife in the Canary Islands), the probability of an alien species thriving, when it has a combination of fitness traits superior to any native species, is obviously much higher on small isolated islands and in hypersaline isolated biotopes, which necessarily display much less variation, than in large continents with large populations and high biodiversity [36].

Also, in the case of *A. franciscana*, in its role as an introduced species, it may well show a similar or higher accumulation of diversity than native American populations. This may result from a combination of multiple local introductions of several origins (anthropic, birds) and numerous translocations from these sites of introduction, or have other origins like local mutations that have no negative effects on individual fitness or as a result of a small population size allowing the accumulation of deleterious mutations (Muller’s Ratchet effect) [38].

## Conclusion

Native *Artemia* in the Canary Islands need to be protected against the invasive *A. franciscana* already present in El Médano pool (Tenerife). In answer to Simberloff’s question about eliminating an invasion or living with it [39], with approaches relying on successful management projects, this invasive population should be eradicated, despite the general pessimism regarding the prospects of eradicating invasive species, which usually ends in managing them at acceptably low densities. Eradication projects do not need to rely on high-tech methods based on sophisticated science or on crude scorched-earth approaches. It should be feasible to reach an economical and socially acceptable solution simply by changing El Médano, a closed and temporary pool, into an open pool with continuously flowing sea water, whereby low sea water salinity and predators should eradicate the invasive species. Local environmental authorities have been made aware of the problem, of the foreseeable outcome and of the above suggested solution.

## Competing interests

The authors declare that they have no competing interests.

## Authors’ contributions

LB, FA and IV prospected the saltworks in the region and collected *Artemia* cyst samples. ER and MM developed laboratory cultures of *Artemia* populations under standard conditions. FA and FH conceived the importance and opportunity of the revision. FH performed statistical discriminant analysis. JCN helped in the design and reviewed the draft and final manuscript. All authors read and approved the final manuscript.

## References

[B1] Engler A Versuch einer Eintwicklungsgesichte, insbesondere der Florengebeite seit der Tertiärperiode. I. Die extra-tropischen Gebeite der nördlischen Hemisphäre 1879 Leipzig: Engelmann

[B2] Bramwell D Kunkel G, Junk W The endemic flora of the Canary Islands: distribution, relationships and phytogeography Biogeography and ecology in the Canary Islands 1976 Netherlands: The Hague

[B3] Geldmacher J Hoernle N van der Bogaard P Zankl G Garbe-Schönberg D Earlier history of the 70-Ma-old Canary hotspot based on the temporal and geochemical evolution of the Selvagem Archipelago and neighbouring seamounts in the eastern North-Africa J Volcanol Geoth Res 2001 111 55 87 10.1016/S0377-0273(01)00220-7

[B4] Mitchell-Thomé RC Outline of the geology of the Cape Verde archipelago Geol Rundsch 1972 61 3 1087 1109 10.1007/BF01820907

[B5] Marin C Luengo A El Jardín de la Sal 1994 de Lanzarote: Publicaciones del Excmo

[B6] VanStappen G Abatzopoulos TJ, Beardmore JA, Clegg JS, Sorgeloos P Zoogeography Artemia. Basic and Applied Biology 2002 Dordrecht: Kluwer Academic Publishers 171 224

[B7] Amat F Cohen RG Hontoria F Navarro JC Further evidence and characterization of *Artemia franciscana* (Kellogg, 1906) populations in Argentina J Biogeogr 2004 31 1735 1749 10.1111/j.1365-2699.2004.01123.x

[B8] Clark LS Bowen ST The genetics of *Artemia salina*. VII. Reproductive isolation J Hered 1976 67 385 388 1021596 10.1093/oxfordjournals.jhered.a108758

[B9] Abatzopoulos TJ Amat F Baxevanis AD Belmonte G Hontoria F Maniatsi S Moscatello S Mura G Shadrin NV Updating geographic distribution of *Artemia urmiana* Günther, 1890 (Branchiopoda, Anostraca) in Europe: An integrated and interdisciplinary approach Internat Rev Hydrobiol 2009 94 5 560 579 10.1002/iroh.200911147

[B10] Pilla EJS Beardmore JA Genetic and morphometric differentiation in Old Worl bisexual species of *Artemia* (the brine shrimp) Heredity 1994 73 47 56 10.1038/hdy.1994.97

[B11] Amat F Persoone G, Sorgeloos P, Roels O, Jaspers E Differentiation in *Artemia* strains from Spain The brine shrimp Artemia. Vol. 1. Morphology, Genetics, Radiobiology, Toxicology 1980 Wetteren: Universa Press 19 39

[B12] Kaiser H Gordon AK Paulet TG Review of the African distribution of the brine shrimp genus *Artemia* Water, SA 2006 32 4 597 604 Available on website http://www.wrc.org.za.

[B13] Amat F Diferenciación y distribución de las poblaciones de *Artemia* de España. VI. Biogeografía Inv Pesq 1983 47 2 231 240

[B14] Varó I Caracterización de dos poblaciones de Artemia parthenogenetica procedentes del archipiélago canario. Estudio comparativo. Tesis de Licenciatura 1988 Santa Cruz de Tenerife: Universidad de La Laguna

[B15] Hontoria F Amat F Morphological characterization of adult *Artemia* (Crustacea, Branchiopoda) from different geographical origin. Mediterranean populations J Plank Res 1992 14 7 949 959 10.1093/plankt/14.7.949

[B16] Ballell L Caracterización de las poblaciones de Artemia (Crustacea, Branchiopoda) presentes en la República de Cabo Verde. Tesis del Master Internacional en Acuicultura 2006 España: Universidad de Las Palmas de Gran Canaria

[B17] Sakai AK Allendorf FW Holt JS Lodge DM Molofsky J With KA Baughman S Cabin RJ Cohen JE Ellstrand NC Mccauley DE O’Neil P Parker IM Thompson JN Weller G The population biology of invasive species Annu Rev Ecol Syst 2001 32 305 332 10.1146/annurev.ecolsys.32.081501.114037

[B18] Mooney HA Cleland EE The evolutionary impact of invasive species Proc Nat Acad Sci USA 2001 98 5446 5451 10.1073/pnas.091093398 11344292 PMC33232

[B19] Williams WD Environmental threats to salt lakes and the likely status of inland saline ecosystems in 2025 Environ Conserv 2002 29 2 154 167

[B20] Amat F Hontoria F Navarro JC Vieira N Mura G Biodiversity loss in the Genus *Artemia* in the Western Mediterranean Region Limnetica 2007 26 177 194

[B21] Mura G Kappas I Baxevanis A Moscatello S D’Amico Q Medina G Hontoria F Amat F Abatzopoulos T Morphological and Molecular data reveal the presence of the invasive *Artemia franciscana* in Margherita di Savoia salterns (Italy) Internat Rev Hydrobiol 2006 91 539 554 10.1002/iroh.200610904

[B22] Amat F Barata C Hontoria F Navarro JC Varó I Biogeography of the genus *Artemia* (Crustacea, Branchiopoda, Anostraca) in Spain Int J Salt Lake Res 1995 3 175 190

[B23] Muñoz J Pacios F Global biodiversity and geographical distribution of diapausing aquatic invertebrates: the case of the cosmopolitan brine shrimp *Artemia* (Branchiopoda, Anostraca) Crustaceana 2010 83 4 465 480 10.1163/001121610X489449

[B24] Triantaphyllidis GV Abatzopoulos TJ Sorgeloos P Review of the biogeography of the genus *Artemia* (Crustacea, Anostraca) J Biogeogr 1998 25 213 226 10.1046/j.1365-2699.1998.252190.x

[B25] Green AJ Sánchez MI Amat F Figuerola J Hontoria F Hortas F Dispersal of invasive and native brine shrimp *Artemia* (Anostraca) via waterbirds Limnol Oceanogr 2005 50 737 742 10.4319/lo.2005.50.2.0737

[B26] Sánchez MI Green AJ Amat F Castellanos EM Transport of brine shrimps via the digestive system of migratory waders: dispersal probabilities depend on diet and season Mar Biol 2007 151 1407 1415 10.1007/s00227-006-0577-9

[B27] de García-Rey E On the distribution of regular winter visitor bird species to the south of Tenerife (Canary Islands) Vieraea 2006 34 25 32

[B28] del Fernández-Castillo M Chorlitejo patinegro en Tenerife: ¿punto y final? Quercus 2010 289 16 22

[B29] Ehrlich PR Mooney HA, Drake JA Which animal will invade? Ecology of Biological Invasions of North America and Hawaii 1984 New York: Springer 9 95

[B30] Lodge DM Biological Invasions: lessons for ecology Trends Ecol Evol 1993 8 133 137 10.1016/0169-5347(93)90025-K 21236129

[B31] McMahon RF Evolutionary and physiological adaptations of aquatic invasive animals: r selection versus resistance Can J Fish Aquat Sci 2002 59 1235 1244 10.1139/f02-105

[B32] Browne RA Competition experiments between parthenogenetic and sexual strains of the brine shrimp *Artemia salina* Ecology 1980 61 3 471 471 10.2307/1937409

[B33] Browne RA Moller V Forbes VE Depledge MH Estimating genetic and environmental components of variance using sexual and clonal *Artemia* J Exp Mar Biol Ecol 2002 267 107 119 10.1016/S0022-0981(01)00363-X

[B34] Barata C Hontoria F Amat F Browne R Competition between sexual and parthenogenetic *Artemia*: temperature and strain effects J Exp Mar Biol Ecol 1996 196 313 328 10.1016/0022-0981(95)00137-9

[B35] Amat F Hontoria F Ruiz O Green A Sánchez M Figuerola J Hortas F The American brine shrimp as an exotic invasive species in the Western Mediterranean Biol Invasions 2005 7 37 47 10.1007/s10530-004-9634-9

[B36] Sax DF Brown JH The paradox of invasion Global Ecol Biogeogr 2000 9 363 371 10.1046/j.1365-2699.2000.00217.x

[B37] Allendorf FW Lundquist LL Population Biology, Evolution, and Control of Invasive Species Conserv Biol 2003 17 1 24 30 10.1046/j.1523-1739.2003.02365.x

[B38] Vidal O García-Berthou E Tedesco PA García-Martín JL Origin and genetic diversity of mosquitofish (*Gambusia holbrooki*) introduced to Europe Biol Invasions 2010 12 841 851 10.1007/s10530-009-9505-5

[B39] Simberloff D We can eliminate invasions or live with them. Successful management projects Biol Invasions 2009 11 149 157 10.1007/s10530-008-9317-z

